# Antifouling Properties of *N*,*N*′-Dialkylated Tetraazamacrocyclic Polyamines and Their Metal Complexes

**DOI:** 10.3390/molecules30112368

**Published:** 2025-05-29

**Authors:** Mathieu Berchel, Dorsaf Malouch, Maryline Beyler, Maryline Fauchon, Yannick Toueix, Claire Hellio, Paul-Alain Jaffrès

**Affiliations:** 1 Laboratoire Chimie Electrochimie Moléculaire Chimie Analytique (CEMCA), UMR 6521, Centre National de la Recherche Scientifique (CNRS), Faculte des Sciences et Techniques, Univ Brest, 6 Avenue Victor Le Gorgeu, 29238 Brest, Francemaryline.beyler@univ-brest.fr (M.B.); paul-alain.jaffres@univ-brest.fr (P.-A.J.); 2 Institut de Recherche pour le Développement (IRD), Ifremer, Laboratoire des Sciences de l’Environnement MARin (LEMAR), Institut Universitaire Européen de la Mer (IUEM), Centre National de la Recherche Scientifique (CNRS), Univ Brest, 29328 Brest, Franceclaire.hellio@univ-brest.fr (C.H.)

**Keywords:** marine bacteria, polyazamacrocycles, metal complexes, copper, zinc, cyclam, cyclen, surface adhesion

## Abstract

The prevention of biofouling (biological fouling) became a major economic and environmental issue. In the present study, we designed a series of four cyclam and cyclen derivatives with a modulation of their lipophilicity by introducing either two benzyl (Bn) groups or two tetradecyl (C_14_) chains in the structure to produce (Cyclam(Bn)_2_, Cyclam(C_14_)_2_, Cyclen(Bn)_2_ and Cyclen(C_14_)_2_). Additionally, copper (Cu) and zinc (Zn) complexes of each compound were prepared and evaluated as potential antifouling candidates against two models of *Vibrio* species (*V. natriegens* and *V. aestuarianus*). The results highlight that no significant antifouling activity was measured for the metal free polyazamacrocyclic derivatives. However, for the metal complexes, the nature of the cation (Cu^2+^ or Zn^2+^) modulates both the growth and adhesion capacities of the two bacteria. Overall, in most cases, Zn complexes showed better activity than the Cu complexes, revealing the importance of the metal cation. Moreover, in the cyclam series, the anti-adhesion properties could be linked to a biocidal effect while a full anti-adhesion activity was observed in the cyclen series.

## 1. Introduction

Biofouling is commonly defined as the accumulation, over time, of micro- and macro-organisms of plant or animal species on natural or artificial surfaces in contact with water [1]. This undesirable phenomenon is the subject of increasing attention due to the significant growth in world maritime trade and the negative impact this has on many sectors of the economy [2]. In addition to the real costs to the shipping industry (biocorrosion, increased drag and fuel consumption, costly regular maintenance), there are also a number of environmental issues to consider, such as increasing carbon dioxide emission or the spread of invasive species that can disrupt local ecosystems and crowd out native species, resulting in ecological imbalances [3,4]. Thus, the development of effective strategies to prevent or combat biofouling is therefore in great demand.

The use of toxic antifouling products is an approach historically used to prevent and limit biofilm formation [5]. Typically, tin-based products such as tributyltin (TBT) have been widely used as a main biocide in antifouling coatings for four decades. However, its high toxicity toward aquatic species resulted in a global ban of TBT-based coatings in 2008 (IMO, 2001) [6,7]. As a direct result of the ban of TBT, the antifouling market witnessed an increased use of copper (Cu) derivatives as a main biocide [8,9], even though Cu was already used before for such applications in paints or as a co-biocide in some TBT paints [10]. Today, copper oxide (Cu_2_O), copper isothiocyanate (CuSCN) and organic copper complexes (Cu-pyrithione) are widely used as primary antifoulants in coatings [8]. Zinc oxides (e.g., ZnO) are also used as biocidal pigments or as booster biocides in combination with Cu to increase its effectiveness and/or to control the polishing rates of the coating [11,12]. The main drawback of such strategies is the risk of the bioaccumulation of these metals in the environment related to their excessive use as antifouling agents. Moreover, heavy metals and biocides can have deleterious effects on non-target organisms or can induce resistance in bacteria [12,13].

The use of metal complexes as an antimicrobial agent is well documented in the literature [14]. Metal complexes could deliver active ions while bypassing the defense mechanisms of microorganisms. Indeed, such compounds offer a possibility to modulate the structure that could enhance the antimicrobial activity of the compound compared to the cationic metal [15], mainly by increasing its lipophilicity. Thus, since the biocidal activity of Cu complexes arises from Cu^2+^ ions, the stability of the chelate must be carefully adjusted in order to ensure the release of adequate quantities of Cu^2+^ while having minimizing toxicity. Cu or Zn pyrithione was introduced as an alternative to TBT in antifouling coatings [16,17]. Although their effectiveness has been proven, several studies have recently addressed their negative impact on the environment as a result of their intensive use [18].

To date, the marine antifouling properties of metal complexes remain poorly investigated [15,19,20,21,22]. In this field of application, macrocyclic polyamines can be considered as potential candidates for formulation in antifouling coatings. Indeed, these ligands and their metal complexes exhibited many biological properties such as antiviral [23], antibacterial [24] and antifungals activities [25,26]. Tetraazamacrocycles and especially cyclam (1,4,8,11-tetraazacyclotetradecane) derivatives complex Cu^2+^ with high selectivity and enhance inertness toward demetallation [27,28], which is a key point for limiting the contamination of marine environments by metals. Interestingly, the high stability of the complex is not necessarily an obstacle to its effectiveness. Some works report on the antimicrobial activity of tetraazamacrocycles complexed with Cu^2+^ [29,30,31]. For example, Hubin et al. have recently reported on the antileishmanial potential of a series of polyazamacrocyles and their metals complexes [32]. Fe^2+^ and Mn^2+^ complexes of a dibenzyl cyclen derivative were proved to be very efficient with moderate cytotoxicity. Also, the antimalarial properties of metal complexes of cross-bridged cyclam and cyclen derivatives have also been reported [33]. The activities of these complexes were found to be highly dependent on the nature of the metal and the size of the macrocycles, and in particular on the stability of the complex Mn^2+^ > Fe^2+^ > Cu^2+^v >> Ni^2+^, Co^2+^, Zn^2+^.

*Vibrios* are a group of common Gram-negative rod-shaped bacteria naturally present in various estuarine, marine and freshwater aquatic environments. *Vibrios* can persist free-living, colonize fishes and marine invertebrates, or be associated with planktons, algae, and abiotic detritus. The ability of *Vibrio* to form biofilms on biotic and abiotic surfaces plays an essential role in their persistence in the environment, and moreover some species of *Vibrio* are pathogens [34]. Thus, some species of the genus *Vibrio* cause significant threats to aquaculture; they affect production and can induce economic losses and public health risks, especially in the context of the use of antimicrobials. Some studies have shown that *Vibrio* biofilms are more resistant to antimicrobial agents and the host’s immune system [35].

**Figure 1:**
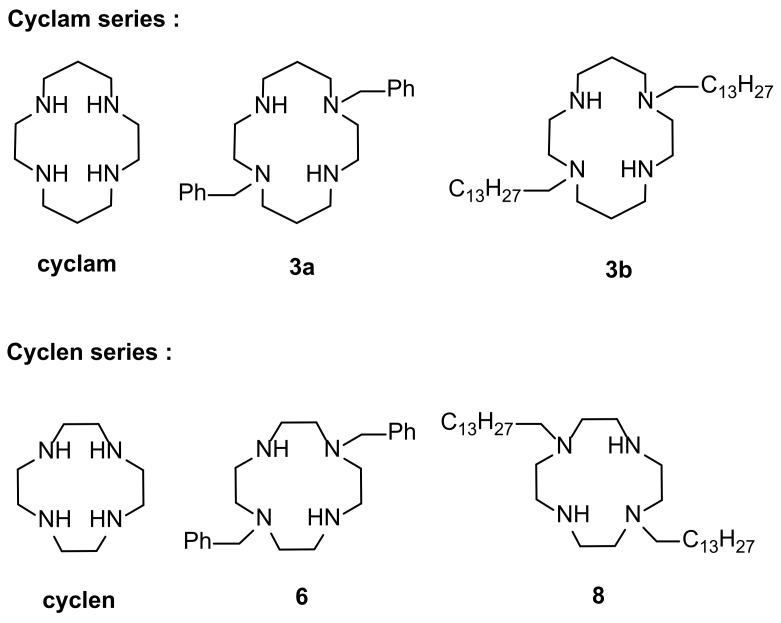
Structure of cyclam and cyclen ligands used in this study.

In the present study, we designed and synthesized two series of *N*,*N′*-*trans*-disubstituted tetraazamacrocyclic polyamines derived either from cyclam or cyclen (1,4,7,10-tetraazacyclododecane). Benzyl and tetradecyl groups were used to modulate the hydrophobic character of these ligands (Figure 1). Thereafter, their copper and zinc complexes were also prepared. Two bacterial strains of the *Vibrio* species (*V. natriegens* and *V. aestuarianus*) were selected, and their adhesion and growth properties were studied. Finally, we also assessed the potential toxicity of the tested compounds on a non-target organism, *Skeletonema costatum*, a marine microalga, representative of species found in coastal temperate environments and a key element of the food-chain.

## 2. Results

### 2.1. Synthesis of Polyazamacrocyclic Compounds

**Scheme 1:**
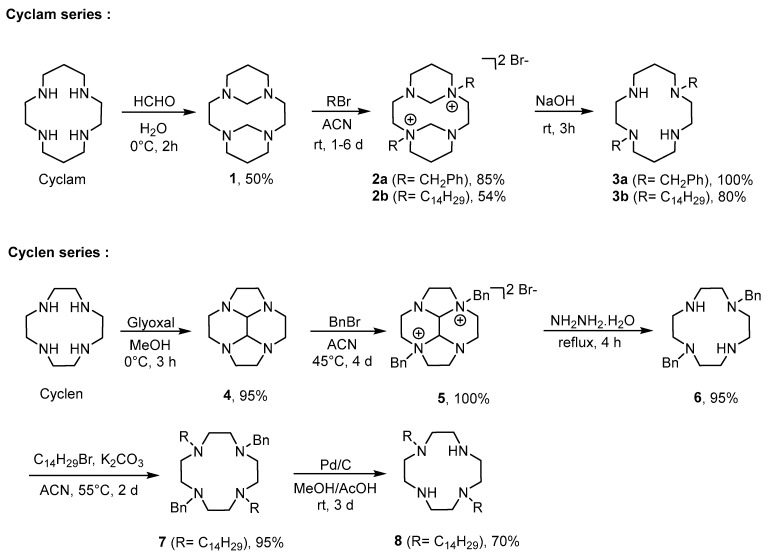
Structure of cyclam and cyclen ligands used in this study.

The synthetic routes used to synthesize cyclam- and cyclen-based ligands are shown in Scheme 1. In the cyclam series, compound **2a** and **2b** were prepared from cyclam bisformyl **1** according to literature procedures [36]. This intermediate allows the preparation of *trans N*,*N*′-disubstituted cyclams with good yields and at room temperature. Noteworthily, in the case of compound **2b,** extending the reaction time to six days was necessary due to the lesser reactivity of 1-bromotetradecane. Diammonium salts **2a** and **2b** were isolated by precipitation. After hydrolysis of the bis-aminal protective groups in aqueous NaOH 3.0 M, compound **3a** and **3b** were obtained in 100% and 80% yields, respectively [36]. In the cyclen series, compound **6** [37] was synthesized according to literature procedures before being alkylated with a slight excess of 1-bromotetradecane in acetonitrile (ACN) and potassium carbonate as a base. The resulting tetra-alkylated product **7** was finally submitted to hydrogenation with Pd/C as a catalyst for three days to furnish the target compound **8** in 70% yields over the two steps.

### 2.2. Synthesis of Copper and Zinc Complexes of Polyazamacrocyclic Compounds

**Scheme 2:**
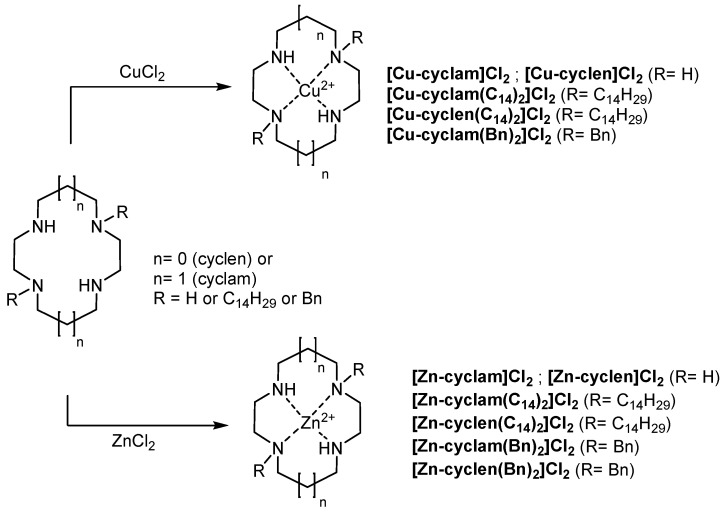
Synthetic routes for the synthesis of metal complexes of cyclam- and cyclen-based ligands used in this study.

Subsequently, metal complexes of the tetraazamacrocycles were prepared as depicted in Scheme 2. Copper(II) chloride (CuCl_2_) and zinc(II) chloride (ZnCl_2_) were used as salts. Briefly, the non-functionalized Cu/Zn-Cyclam and Cu/Zn-Cyclen complexes were synthesized in hot water and isolated quantitatively. In the case of the *N*-benzylated Cu/Zn-Cyclen(Bn)_2_ complexes, ligands were dissolved in ACN and an aqueous solution of metal salt was added. The corresponding complexes were isolated by precipitation with moderate yields (65% and 43%, respectively). Due to their low solubility in water, *N*alkylated Cu/Zn-Cyclam(C14)_2_ was synthesized in organic solvent (MeOH or DCM/MeOH mixture) at room temperature or with slight heating. Targeted compounds were isolated in high yields (90%).

### 2.3. Effect of CuCl_2_ and ZnCl_2_ on Bacterial Growth and Adhesion

First of all, a growth kinetic was performed for each bacterial strain over a period of 24 h after exposition to CuCl_2_ or ZnCl_2_ (see Supplementary Materials). Three concentrations of each salt were assessed: 2, 20 and 200 μM. The kinetics curves did not highlight any significant effect at 2 or 20 μM regardless of the strain tested. However, at the highest concentration tested (200 μM), a shift in the curve was recorded, and growth started at 17.5 h for *V. natriegens* in the presence of ZnCl_2_, highlighting the sensitivity of this strain toward this salt. No growth inhibition was observed with CuCl_2_ for *V. natriegens*. Considering *V. aesturianus*, this strain was less sensitive to metal salts; only a minor growth delay was observed for CuCl_2_, although the OD curve reached the control values after 24 h. These results show that the two strains respond differently to copper and zinc salts but almost no toxicity was observed at such concentrations. The concentration of 200 μM was selected for further investigations. Table 1 shows the growth and adhesion inhibition percentage of the two bacterial strains for CuCl_2_ and ZnCl_2_ at 200 μM. As mentioned previously, *V. natriegens* is more sensitive to ZnCl_2_ since its growth is inhibited by up to 60%. This growth inhibition is well correlated with an inhibition in adhesion capacities. However, this strain seemed to be less sensitive to CuCl_2_ (37.5% ± 9.5 percentage of growth inhibition), and no effect was measured on adhesion (Table 1). In contrast, the growth of *V. aestuarianus* was inhibited by CuCl_2_ up to 56% (Table 1), while no effect was observed on either growth or adhesion with ZnCl_2_ in accordance with the growth kinetics.

### 2.4. Effect of Free Base Ligands on Bacterial Growth and Adhesion

Thereafter, the effect of the free base ligands on bacterial strains was also considered (Table 1). Generally, neither bacterial growth nor adhesion are significantly inhibited by free base ligands regardless of the type of macrocycle. Surprisingly, the alkylation of the macrocycle with two benzyl groups leads to an increase in growth inhibition to 52.9% ± 1.8 for cyclam(Bn)_2_ and 40% ± 3.4 for cyclen(Bn)_2_ for *V. natriegens*. This effect is correlated with a slight decrease in bacterial adhesion capacities in the presence of cyclam(Bn)_2_. On the other hand, no notable effect was observed for *V. aesturianus* regardless of the ligand tested (Table 1).

### 2.5. Effect of Copper and Zinc Complexes on Bacterial Growth and Adhesion

The antibacterial activity of Cu and Zn complexes was evaluated against the two bacterial strains at 200 μM (Table 1). Cu complexes ([Cu-cyclam]Cl_2_ and [Cu-cyclen]Cl_2_) showed no effect on the bacteria regardless of the ligands tested. These results are in accordance with the stability described for such polyamines. Interestingly, Cu complexes are tolerated by the bacteria, especially the strain *V. aestuarianus*, which was previously sensitive to CuCl_2_ at 200 μM. The introduction of two tetradecyl chains on the cyclam was considered to produce an amphiphilic Cu complex. We hypothesized that an amphiphilic compound would interact more easily with the membrane of the bacteria to alter its integrity. However, no improvement in the growth inhibition of the bacterial strains was observed with compound [Cu-cyclam-(C_14_)_2_]Cl_2_. Similarly, the addition of Cu complexes had no influence on the adhesion capacities of the bacterial strains regardless of the compounds tested. Table 1 also shows that the two *Vibrio* species are more sensitive to Zn complex series. While no significant growth or adhesion inhibition was measured with [Zn-cyclam]Cl_2_, the functionalization of the ligand with two tetradecyl chains [Zn-cyclam-(C_14_)_2_]Cl_2_ or two benzyl groups ([Zn-cyclam(Bn)_2_]Cl_2_) led to a significant adhesion inhibition for *V. natriegens* of 82.8% ± 6.9 and 92.0% ± 7.3, respectively. For each of these compounds, the adhesion inhibition seemed to be related to a toxic effect, since an almost complete growth inhibition was observed (99.0% ± 0.3 for [Zn-cyclam(C_14_)_2_]Cl_2_) and 99.6% ± 3.3 for [Zn-cyclam(Bn)_2_]Cl_2_). For *V. aestuarianus*, a different trend was observed.

At 200 μM, compound [Zn-cyclam(C_14_)_2_]Cl_2_ induced a moderate adhesion inhibition of 51.1% ± 19.9, while no effect on growth was observed. Conversely, [Zn-cyclam(Bn)_2_]Cl_2_ led to a reduction in growth (74.0 ± 5.7) with no effect on adhesion. This suggests a biocidal effect of compound [Zn-cyclam(Bn)_2_]Cl_2_ on this strain. In the cyclen series, two compounds showed an interesting adhesion inhibition capacity on the strain *V. aestuarianus*. Indeed, [Zn-cyclen]Cl_2_ and [Zn-cyclen(C_14_)_2_]Cl_2_ led to an adhesion inhibition percentage of 41.8% ± 14.8 and 61.3% ± 14.6, respectively (Table 1), with no biocidal effect. Later, no effect was observed for compound [Zn-cyclen(Bn)_2_]Cl_2_ regardless of the bacterial strain tested.

### 2.6. Effect of Copper and Zinc Complexes on the Microalgae S. costatum

The effect of the most promising complexes was further investigated regarding their toxicity against the microalgae *S. costatum*. Table 2 summarizes the growth percentage for three concentrations (2, 20 and 200 μM) of compounds (CuCl_2_, ZnCl_2_ and Cu/Zn complexes) after 96 h of exposure. First, all the compounds tested were tolerated by the microalgae for concentrations up to 20 μM. At 200 μM, a different trend appeared, and the microalgae showed a great sensitivity toward CuCl_2_ while ZnCl_2_ did not produce any toxicity at these concentrations. Additionally, all the ligands and Zn complexes tested caused a total growth inhibition of the microalgae at 200 μM, except for compound [Zn-cyclen]Cl_2_. It appears that the increase in the lipophilic nature of the ligands increased the toxicity towards *S. costatum*.

## 3. Discussion

Tetraazamacrocyclic polyamines possess numerous biological properties [38,39]. Over the past years, they have given rise to growing interest in medicine, especially as contrast agents for imaging (MRI, TEP) [40]. Such polyamines are potentially good candidates for developing antifouling agents based on metal complexes considering their biocompatible nature. They are also known to form highly stable metal complexes with copper, thus offering the possibility of competing with natural seawater ligands and metals [30,41]. To investigate the antibiofilm potential of tetraazamacrocycle-based metal complexes, two kinds of macrocycles were selected as organic platforms (Figure 1). For each series, a set of three ligands (cyclam series and cyclen series) was designed and their copper and zinc complexes were synthesized. In addition to the starting polyamines (amine free base), *N*,*N*-dibenzyl and *N*,*N*-ditetradecyl ligands were designed in order to modulate the hydrophobic character of the ligands. We postulated that benzylated ligands would be more hydrophobic than the non-alkylated compounds, and that the introduction of two tetradecyl chains would give the ligands an amphiphilic character. In addition, we hypothesized that the introduction of two tetradecyl chains would promote the interaction of the compounds with the membrane of the bacteria. The rationale was that biocidal effects could result from both the destabilization of the membrane (hydrophobic and electrostatic interactions) and the release of biocidal metallic cations near or inside the bacteria [42,43]. Furthermore, the *N* alkylation of the macrocycle could lead to modulation in the stability of the complex (vs. free amine ligand) and subsequently influence the metal release kinetics. In this study, we first investigated the antifouling properties of copper (CuCl_2_) and zinc (ZnCl_2_) salts, and the antifouling properties of free base ligands against two selected bacterial strains of *Vibrio* species (*V. natriegens* and *V. astuarianus*). Three concentrations were tested for each compound (2, 20 and 200 μM). Obviously, only the highest concentration of 200 μM significantly impaired bacterial growth and adhesion. In addition, the effect of salts depended on the bacterial strain tested. While the growth of *V. natriegens* was inhibited by ZnCl_2_ (up to 60%) and its adhesion capacities affected, *V. aestuarianus* was more sensitive to CuCl_2_ (up to 56%). Conversely, *V. natriegens* was not sensitive to CuCl_2_, and the same trend was observed for *V. aestuarianus* against ZnCl_2_. For non-substituted ligands, no significant inhibition of growth and adhesion was observed regardless of the bacteria tested except for benzylated derivatives against *V. natriegens* (growth inhibition: 52.9% ± 1.8 for cyclam(Bn)_2_ and 40% ± 3.4 for cyclen(Bn)_2_). Interestingly, all the ligands were tolerated by the two microorganisms under our conditions (Table 1). Thereafter, the antifouling potential of metal complexes of the tetraazamacrocyclic ligands was considered. Ligands with benzyl or aliphatic chains (tetradecyl chains) were designed with the aim of modulating the amphiphilic character of the compounds, and in the case of the alkyl chains, to promote their insertion into the external membrane of bacteria. The cationic nature of the metal complexes could also contribute to the antimicrobial properties of the compounds. The results showed that with copper, none of the complexes tested led to a significant growth or adhesion inhibition for the two bacterial strains. The results suggest that the complexes formed in the presence of CuCl_2_ ([Cu-cyclam]Cl_2_, [Cu-cyclen]Cl_2_ and [Cu-cyclam-(C_14_)_2_]Cl_2_) are sufficiently stable, and the assumption of a partial release of the cation in the medium does not cause any toxicity. Thus, only these three copper derivatives were considered in this study. However, the results showed a more important effect of zinc complexes, especially for N,N-dialkylated complexes. Indeed, the insertion of benzyl groups or tetradecyl chains into zinc complexes led to a biocidal effect against *V. natriegens*, which resulted in increased inhibition of growth and adhesion capacities. For *V. aestuarianus*, a less general trend was observed. Basically, [Zn-cyclam(C_14_)_2_]Cl_2_ reduced the adhesion of the bacteria with no effect on growth, while compound [Zn-cyclam(Bn)_2_]Cl_2_ induced a biocidal effect (growth inhibition 74.0% ± 5.7). Surprisingly, in the cyclen series, compounds [Zn-cyclen]Cl_2_ and [Zncyclen(C_14_)_2_]Cl_2_ showed an interesting inhibition of adhesion (41.8% ± 14.8 and 61.3% ± 14.6 respectively, Table 1), without any biocidal effect. These results suggest that increasing the amphiphilic nature of the Zn-cyclam derivatives could be a convenient strategy to modulate their potential as biocides. Interestingly, introducing aliphatic chains on Zn-cyclam or Zn-cyclen helped to improve anti-adhesion properties with the strain *V. aestuarianus*. Finally, the cytotoxicity of the compounds was evaluated against a model of microalgae *S. costatum*. Indeed, safety issues are a critical parameter to consider when developing new antifouling agents in order to limit side effects on non-target micro- and macro-organisms or the appearance of resistance. Table 2 shows that after 96 h of exposure, no cytotoxic effect was observed up to 20 μM for any metal salts or the most promising complexes. However, at 200 μM, a complete biocidal effect was measured except for ZnCl_2_ and [Zn-cyclen]Cl_2_. Objectively, these series of compounds deserve to be investigated in more detail in the future for the development of antifouling agents.

## 4. Materials and Methods

All chemicals were purchased from Sigma-Aldrich (St Quentin Fallavier, France) or Acros-Fischer (Noisy-le-Grand, France). Cyclam and cyclen free bases were purchased from Chematech (Dijon, France). Solvents were dried prior to use with a solvent purification MBraun-SPS system (MBRAUN, Merignac, France). All compounds were characterized by NMR analysis (^1^H, ^13^C) at the “Service communs” of the University of Brest and recorded on Bruker 300 (AMX-3 300), Bruker 400 (Avance DRX 400) or 500 MHz (Avance DRX 500) spectrometers (BRUKER France SAS, Palaisseau, France). Mass spectrometry measurements (HRMS Q-Tof MaXis instrument equipped with ESI, APCI, APPI and nano-ESI sources) were performed at the Institute of Organic and Analytic Chemistry—ICOA in Orléans, France (WATERS France, Guyancourt, France). Compounds **3a** [39] and **6** [41] were synthesized according to previously described methods.

### 4.1. Preparation of Compound ***3b***

Cyclam bisformyl **1** (500 mg, 2.23 mmol) was dissolved in 15 mL of freshly distilled acetonitrile. 1-bromotetradecane (2.7 mL, 8.91 mmol) was added and the solution was vigorously stirred for 6 days at room temperature. The precipitate was filtered, washed with acetonitrile and dried to furnish **2b** as a white solid (940 mg, 54%). Crude compound **2b** (940 mg, 1.21 mmol) was suspended in aqueous NaOH 3.0 M (33 mL). The mixture was stirred at room temperature for 4 h. A white sticky paste was formed. DCM (50 mL) was added to dissolve the cake and the organic was washed with water (3 × 30 mL), dried over MgSO4, filtered and concentrated to yield compound **3b** (897 mg, 80%). ^1^H RMN (300 MHz, CDCl_3_): δ 2.92 (bs, 4 H), 2.80–2.66 (bs, 6 H), 2.57–2.45 (m, 8 H), 1.90–176 (m, 2 H), 1.48 (bs, 4 H), 1.27 (bs, 52 H), 0.89 (m, 8H). ^13^C RMN (75 MHz, CDCl_3_): δ 54.4, 51.1, 51.0, 50.1, 48.2, 47.1, 31.8, 29.6, 29.4, 29.3, 27.5, 27.3, 26.0, 24.2, 22.8, 22.5, 14.0. HRMS (ESI positive) *m*/*z* calcd. for [C_38_H_80_N_4_ + H]^+^: 593.6455, found: 593.6454, [M + H]^+^.

### 4.2. Preparation of Compound ***7***

Cyclen dibenzyl **6** (280 mg, 0.79 mmol) was dissolved in freshly distilled acetonitrile. K_2_CO_3_ (450 mg, 3.16 mmol) and 1-bromotetradecane (0.50 mL, 1.66 mmol) were added. The reaction was stirred at 55 °C for 2 days. After cooling to room temperature, the reaction was filtered. The solid was taken up in DCM and filtered. The filtrate was evaporated to dryness to give compound **7** as a brownish oil (515 mg, 95%). ^1^H RMN (400 MHz, CDCl_3_): δ 7.44 (dd, 4 H, ^3^*J* = 6.9 Hz, ^4^*J*= 1.6 Hz), 7.34 (td, 4 H, ^3^*J* = 7.4 Hz, 4*J* = 1.2 Hz), 7.26 (m, 2 H), 3.58 (s, 4 H), 2.68 (m, 16 H), 2.26 (m, 4 H), 1.29 (m, 56 H), 0.92 (m, 6 H). ^13^C RMN (125 MHz, CDCl_3_): δ 142.6, 131.8, 130.7, 129.3, 63.1, 58.5, 55.5, 55.1, 34.6, 32.5, 32.4, 32.2, 30.4, 25.4, 16.9. HRMS (ESI positive) *m*/*z* calcd. for [C_50_H_88_N_4_ + H]^+^: 745.7082, found: 745.7079, [M + H]^+^.

### 4.3. Preparation of Compound ***8***

Compound **7** (515 mg, 0.75 mmol) was dissolved in a mixture of MeOH/AcOH (10 mL/0.5 mL). Pd/C (100 mg, 0.94 mmol) was added. The mixture was degassed, placed under hydrogen atmosphere (1 atm) and stirred at room temperature for 3 days. The catalyst was filtered off over a Celite^®^ pad (Sigma-Aldrich, St Quentin Fallavier, France). The filtrate was evaporated under reduced pressure. The solid was taken up in DCM and washed with aqueous NaOH 3.0 M (1 × 10 mL) and H_2_O (2 × 10 mL). The organic phase was dried over MgSO_4_, filtered and evaporated to dryness, affording compound **8** as a yellow oil (313 mg, 74%). ^1^H NMR (500 MHz, CDCl_3_): δ 2.59 (m, 8 H), 2.50 (m, 8H), 2.37 (t, 4 H, ^3^*J* = 7.2 Hz), 1.44 (m, 4 H), 1.25 (m, 44 H), 0.86 (t, 6 H, ^3^*J* = 6.8 Hz). ^13^C NMR (125 MHz, CDCl_3_): δ 57.8, 54.9, 48.0, 34.6, 32.5, 32.4, 32.4, 32.1, 30.4, 30.3, 30.2, 30.1, 25.4, 16.8. HRMS (ESI positive) *m*/*z* calcd. for [C_36_H_76_N_4_ + H]^+^: 565.6143, found: 565.6146, [M + H]^+^.

### 4.4. General Procedure for Synthesis of Cu and Zn Complexes from Free Base Ligands

The free-base macrocycle cyclam or cyclen (1.0 equiv.) was dissolved in deionized water (10 mL). CuCl_2_ or ZnCl_2_ (1.0 equiv.) dissolved in deionized water (5 mL) was added to the previous solution, and the reaction was heated to 80 °C overnight. Thereafter, the solvent was removed by evaporation under reduced pressure to quantitatively give the desired complex.

[Cu-cyclam]Cl_2_. From cyclam (100 mg, 0.50 mmol) and CuCl_2_ (67.1 mg, 0.50 mmol). Purple solid (167 mg, 99%). HRMS (ESI positive, H_2_O) *m*/*z* calcd. for [C_10_H_24_N_4_Cu]^2+^, 131.5643; found 131.5645 [M]^2+^.

[Zn-cyclam]Cl_2_. From cyclam (106 mg, 0.53 mmol) and ZnCl_2_ (71.2 mg, 0.53 mmol). White solid (177 mg, 99%). ^1^H NMR (400 MHz, D_2_O): d 3.33 (m, 8 H), 3.17 (m, 4 H), 2.87 (m, 4 H), 2.64 (m, 4 H), 2.04 (m, 2 H), 1.78 (m, 2 H). ^13^C NMR (125 MHz, D_2_O): δ 52.7, 50.5, 30.5. HRMS (ESI positive, H_2_O) *m*/*z* calcd. for [C_10_H_24_N_4_Zn]^2+^, 132.0641; found 132.0640 [M]^2+^.

[Cu-cyclen]Cl_2_. From cyclen (100 mg, 0.58 mmol) and CuCl_2_ (70.0 mg, 0.58 mmol). Blue solid (170 mg, 99%). ESI-HR-MS (positive, H_2_O) *m*/*z* calcd. for [C_8_H_20_N_4_Cu]^2+^, 117.5486; found 117.5490 [M]^2+^.

[Zn-cyclen]Cl_2_. From cyclen (100 mg, 0.53 mmol) and ZnCl_2_ (71.0 mg, 0.53 mmol). White solid (171 mg, 99%). ^1^H NMR (400 MHz, D_2_O): δ 3.81 (bs, 2 H), 3.07 (m, 8 H), 2.95 (m, 8 H). 13C (77 MHz, D_2_O): δ 43.5. HRMS (ESI positive, H_2_O) *m*/*z* calcd. for [C_8_H_20_N_4_ZnCl]^+^, 271.0662; found 271.0658 [MCl]^+^.

### 4.5. General Procedure for Synthesis of Cu and Zn Complexes from ***3b***

Compound **3b** (1.0 equiv., 39.2 mg, 0.066 mmol) was suspended in MeOH (5 mL) and heated to 50 °C. CuCl_2_ or ZnCl_2_ (1.0 equiv.) (9.0 mg, 0.066 mmol) in MeOH (2 mL) was added to the previous solution. The mixture became progressively clear and was reacted at 50 °C overnight. The solvent was removed under reduced pressure and the solid was taken up in DCM and washed with water (2 × 10 mL). The organic phase was concentrated to dryness to give the desired complex.

[Cu-cyclam(C_14_)_2_]Cl_2_. From **3b** (39.2 mg, 0.66 mmol) and CuCl_2_ (9.0 mg, 0.66 mmol). Blue solid (44.2 mg, 92%). HRMS (ESI positive) *m*/*z* calcd. for [C_38_H_80_N_4_Cu]^2+^, 327.7877; found 327.7839 [M]^2+^.

[Zn-cyclam(C_14_)_2_]Cl_2_. From **3b** (40.6 mg, 0.068 mmol) and ZnCl_2_ (9.3 mg, 0.068 mmol). White solid (44.6 mg, 90%). ^13^C NMR (126 MHz, CDCl_3_): δ 57.2, 56.9, 52.6, 51.0, 49.7, 34.6, 32.4, 32.3, 32.1, 30.3, 29.9, 27.1, 25.4, 23.1, 16.8. HRMS (ESI positive, H_2_O) *m*/*z* calcd. for [C_38_H_80_N_4_ZnCl]^+^, 691.5357; found 691.5368 [MCl]^+^.

### 4.6. Synthesis of [Zn-cyclen(C_14_)_2_]Cl_2_

Compound **8** (88 mg, 0.16 mmol) was dissolved in DCM (5 mL). ZnCl_2_ (22 mg, 0.16 mmol) in MeOH (2 mL) was added to the previous solution. The mixture was stirred at room temperature overnight and washed with water (2 × 10 mL). The organic phase was concentrated to dryness to give complex [Zn-cyclen(C_14_)_2_]Cl_2_ as a white solid (98 mg, 90%). ^1^H NMR (400 MHz, D_2_O): δ 2.99 (m, 4 H), 2.77 (m, 14 H), 1.60 (m, 4 H), 1.28 (m, 42 H), 0.89 (t, 6 H). ^13^C NMR (126 MHz, MeOD): δ 55.3, 50.6, 44.5, 33.0, 30.7, 30.7, 30.6, 30.6, 30.5, 30.4, 28.4, 23.7, 23.4, 14.6. HRMS (ESI positive, H_2_O) *m*/*z* calcd. for [C_36_H_76_N_4_ZnCl]^+^, 663.5044; found 663.5040 [MCl]^+^.

### 4.7. General Procedure for Synthesis of Zn Complexes from Cyclam(Bn)_2_ and Cyclen(Bn)_2_

The procedure was adapted from the literature [44]. Briefly, the free base macrocycle was dissolved in acetonitrile (20 mL) and 1 equivalent of ZnCl_2_ dissolved in water (2 mL) was added to the previous solution. The mixture was stirred at room temperature overnight. The precipitate was filtered, washed with acetonitrile and dried.

[Zn-cyclam(Bn)_2_]Cl_2_. From compound **3b** (213 mg, 0.56 mmol) and ZnCl_2_ (80 mg, 0.56 mmol). White solid (125 mg, 43%). ^1^H NMR (500 MHz, d_6_-DMSO/D_2_O): δ 7.80 (m, 6 H), 7.66 (m, 4 H), 4.51 (m, 4 H), 4.58–2.67 (m, 20 H), 2.09 (t, 2 H). ^13^C NMR (125 MHz, d_6_-DMSO/D_2_O): δ 136.8, 136.5, 133.8, 133.7, 60.5, 57.8, 57.4, 55.0, 50.4, 28.3. HRMS (ESI positive, H_2_O) *m*/*z* calcd. for [C_24_H_36_N_4_Zn − H]^+^, 443.2148 ; found 443.2148 [M − H]^+^.

[Zn-cyclen(Bn)_2_]Cl_2_. From compound **6** (134 mg, 0.38 mmol) and ZnCl_2_ (52 mg, 0.38 mmol). White solid (120 mg, 65%). ^1^H NMR (400 MHz, D_2_O): d 7.39 (m, 6 H), 7.33 (m, 4 H), 4.21 (m, 2 H), 3.94 (s, 4 H), 3.15 (m, 4 H), 2.82–2.72 (m, 8 H), 2.62–2.56 (m, 4 H). ^13^C NMR (125 MHz, D_2_O): d 134.7, 134.1, 131.5, 131.4, 59.1, 51.5, 45.6. HRMS (ESI positive, H_2_O) *m*/*z* calcd. for [C_22_H_32_N_4_Zn]^2+^, 208.0954; found 208.0957 [M]^2+^.

### 4.8. Marine Bacteria

The tested strains, *Vibrio aestuarianus* and *Vibrio natriegens* (DSM No.: 19606 and 19623 respectively), were obtained from the Leibniz Institute DSMZ-German Collection of Microorganisms and Cell Cultures GmbH (Brunswick, Germany). *V. aestuarianus* cultures were prepared using marine broth (BD 279110, Fisher Scientific, Illkirch, France), and *V. natriegens* was prepared using nutrient broth (CM0001B, Fisher Scientific, France) supplemented with 1.5% NaCl (Sigma-Aldrich, France).

### 4.9. Bacterial Growth and Adhesion Assays

The assays were run using the protocol of Bovio et al. [45]. Compounds were dissolved in methanol as a carrier solvent. After evaporation, bacterial suspensions (100 μL aliquots, 2 × 108 colony forming units/mL) were aseptically added to the microplate wells containing tested compounds at 2, 20 and 200 μM. The plates were incubated for 24 h at 30 °C. All samples were tested in triplicate. The controls were also tested in triplicate and included bacterial culture and media control wells. Experimental errors were expressed as the standard deviation obtained from three replicates. Bacterial growth was monitored spectroscopically at 620 nm. Growth inhibition percentage (*IP*) was calculated as follows:

$$IP=\left[\frac{\left(C_{620nm}-B_{620nm}\right)-(V_{620nm}-B_{620nm})}{\left(C_{620nm}-B_{620nm}\right)}\right]\times 100$$

where *C*: control OD_620nm_ of bacterial suspension without the tested compound, *B*: OD_620nm_ of culture medium without bacteria, and *V*: OD_620nm_ of the bacteria with the tested compound. After 24 h of incubation time, wells were emptied and rinsed with 100 μL of sterile artificial seawater (100 μL) to remove non-adherent cells. The microplates were air-dried at room temperature. The residual bacterial biofilm was stained with aqueous crystal violet (100 μL, 0.1% Cristal Violet (CV) *v*/*v*) for 30 min. After rinsing the excess of CV with deionized water, the plates were allowed to dry for 48 h at room temperature. An amount of 100 μL of ethanol 80% was added to each well to solubilize the biofilm, and the absorbance measures were monitored at 595 nm. The adhesion inhibition percentage was calculated as follows:

$$IP=\left[\frac{\left(C_{595nm}-B_{595nm}\right)-(V_{595nm}-B_{595nm})}{\left(C_{595nm}-B_{595nm}\right)}\right]\times 100$$

where *C*: control OD_595nm_ of bacterial suspension without the tested compound, *B*: OD_595nm_ of culture medium without bacteria and *V*: OD_595nm_ of the bacteria with the tested compound. Experimental errors were expressed as the standard deviation obtained from three replicates.

### 4.10. Marine Microalga Culture and Growth

*Skeletonema costatum*, a species of marine microalgae, a representative of species found in coastal temperate environments and a key component of the marine food web, was used to carry out tests to assess the potential toxicity of the compounds on non-target organisms. The diatom *Skeletonema costatum* (*S. costatum*) UTEX LB 2308 was obtained from the culture collection of LEMAR (France). Prior to experiments, microalgae were grown in sterile F/2 medium for 7 days at room temperature [46]. The phytoplankton strains were cultivated at a light intensity of 130 mol. Photons were m^−2^.s^−1^ from a cool white fluorescent light, with an 8:16 light/dark photoperiod (Quantometer Li-250 equipped with a spherical sensor). Microalgae were grown in a sterile TC flask T75, standard (Sigma Aldrich, St Quentin Fallavier, France) of 50 mL, filled with 30 mL of sterile culture medium. Microalgae cultures were diluted weekly in order to maintain exponential growth.

### 4.11. Microalga Inoculation

Miniaturized tests were carried out in 96-well polystyrene microplates (Greiner Bio-One GmbH, Ref 655906, untreated, Courtaboeuf, France) [47]. Each well was filled with 100 μL of the compound tested; after the evaporation of the solvent, 100 μL of microalga culture was added to the wells. For each species, after counting the cell density of the initial culture, an intermediate culture was obtained by dilution of the initial culture at a concentration of 200,000 cell·mL^−1^. Then, 0.1 mL of this intermediate culture was added to each well to obtain an initial concentration of 20,000 cell·mL^−1^ at the beginning of exposure. Controls of tested compounds and sterile medium were tested to measure the possible emission of fluorescence due to the compound. The microplates were covered with their lids, allowing the gas exchange to continue. Each concentration was tested in six replicates, and each microplate contained at least 3 controls divided into two conditions: the medium control containing the culture medium usually used for the microalgae and the solvent control condition containing the solvent of the compound. The growth of microalgae was measured every 24 h, between the start of exposure at t = 0, and the end at t = 96 h (±2 h), during the enlightened phase, using fluorescence emitted by chlorophyll pigments. The microplates were read using a SAFIRE microplate reader (TECAN) driven by the XFluor4beta Excel^®^ macro. The excitation/emission wavelengths were 450/684 nm (10 nm bandwidth), nine readings were made per well from below, with an integration time of 20 s. Each microplate was stirred for 20 s before readings using an Orbis Plus microplate agitator (Mikura Ltd.) in orbital mode. For each species and compound tested, in each well, the growth rate μ was calculated over the 96 h exposure, based on the following equation:

$$\mu =ln(F_{t}-F_{0})/t$$

where F_t_ is the fluorescence (arbitrary unit, a.u.) of the well at t-time (h), μ (h^−1^) is the growth rate and F_0_ the initial fluorescence at t = 0 h. For the interpretation of the results, the medium control was considered as a reference. This control can be compared with the solvent control containing the solvent of the tested compound (Methanol). This allows for the assessing of to what extent the use of solvent can affect the growth rate of microalgae.

### 4.12. Statistical Analysis

Experiments were performed in sextuplets and results were obtained from at least two independent tests. Mean values are reported with the standard deviation. All statistical analyses were implemented in R software (R Studio version 3.6.1 (2019-07-05)). A parametric test (i.e., Anova, Dunnett, Tukey) and a non-parametric test (i.e., Kruskal–Wallis, Dunn) (rstatix version 0.7.0, ggplot2 version 3.2.1, multcomp version 1.4–12, car version 3.0–10, dunn.test version 1.3.5) were used to determine the statistical significance of the means between the control and test samples (bacterial growth, biofilm production), with a significance level of 0.05. Excel Regtox macro (version EV7.0.5.xls) was used on dose-response curves to estimate concentrations leading to growth rate inhibition and adhesion inhibition at 50% (EC50) [48].

## 5. Conclusions

To conclude, the present study aims to assess the potential of two series of tetraazamacrocyclic ligands and their metal complexes as potential classes of antifouling compounds. The functionalization of cyclam and cyclen with benzyl groups resulted in moderate activity against *V. aestuarianus* and *V. natriegens*. However, corresponding Zn complexes seemed to be more active. Indeed, Zn complexes, which are known to form less stable complexes with cyclam (and cyclen) compared to their Cu analogues, showed a strong tendency to improve the antifouling property of the ligands when the lipophilic nature of the complex increased. In the context of a high exposure to biocides to fight against biofouling and the appearance of a resistance mechanism in bacteria, we highlight the importance of proposing effective alternative antifouling agents. Among others, tetraazamacrocycles and their metal complexes are promising antifouling alternatives. To get more insight into the antifouling activities of these polyamines and their metal complexes, a more in-depth structure–activity study will be performed in future work.

## Figures and Tables

**Table 1:** Growth and adhesion inhibition of bacteria. Compounds were tested at 200 µM (Mean ± SD, *n* = 3).

Compounds		Growth Inhibition Percentage at 200 µM		Adhesion Inhibition Percentage at 200 µM	
		*V. natriegens*	*V. aestuarianus*	*V. natriegens*	*V. aestuarianus*
Salts	CuCl_2_	37.5 ± 9.5	56.2 ± 0.2	−11.3 ± 7.1	−10.4 ± 5.0
	ZnCl_2_	60.9 ± 8.5	−12.1 ± 4.8	60.6 ± 21.8	20.1 ± 4.7
Ligands	Cyclam	−0.2 ± 8.3	12.2 ± 20.2	−2.0 ± 11.4	0.7 ± 31.2
	Cyclam(Bn)_2_	52.9 ± 1.8	21.0 ± 3.7	32.3 ± 2.9	−20.9 ± 0.5
	Cyclam(C_14_)_2_	6.3 ± 8.5	−21.7 ± 33.9	−54.9 ± 40.9	−19.9 ± 7.4
	Cyclen	−0.6 ± 7.5	−1.4 ± 0.1	−5.7 ± 2.4	14.9 ± 2.5
	Cyclen(Bn)_2_	40.0 ± 3.4	11.6 ± 22.2	−7.5 ± 9.0	1.9 ± 3.7
	Cyclen(C_14_)_2_	−12.9 ± 4.0	20.6 ± 23.2	−124.7 ± 1.1	13.8 ± 12.8
Cu complexes	[Cu(cyclam)]Cl_2_	11.7 ± 7.1	−3.5 ± 11.5	−72.3 ± 22.2	−51.1 ± 23.6
	[Cu-cyclam-(C_14_)_2_]Cl_2_	−8.9 ± 17.2	34.5 ± 7.8	−25.5 ± 9.9	8.2 ± 29.9
	[Cu-cyclen]Cl_2_	9.2 ± 10.7	29.7 ± 0.9	−30.0 ± 13.1	−10.8 ± 3.0
Zn complexes	[Zn-cyclam]Cl_2_	10.6 ± 11.5	−0.2 ± 7.3	−33.9 ± 12.9	4.3 ± 1.9
	[Zn-cyclam(C_14_)_2_]Cl_2_	99.0 ± 0.3	14.3 ± 11.2	82.8 ± 6.9	51.1 ± 19.9
	[Zn-cyclam(Bn)_2_]Cl_2_	99.6 ± 3.3	74.0 ± 5.7	92.0 ± 7.3	−28.0 ± 21.8
	[Zn-cyclen)]Cl_2_	−22,1 ± 7.2	17.5 ± 0.5	−7.4 ± 23.5	41.8 ± 14.8
	[Zn-cyclen(C_14_)_2_]Cl_2_	−11.1 ± 7.1	−13.3 ± 17.6	−3.6 ± 10.8	61.3 ± 14.6
	[Zn-cyclen(Bn)_2_]Cl_2_	25.1 ± 3.4	20.9 ± 4.1	−15.4 ± 5.5	−3.6 ± 8.8

**Table 2:** Growth of *S. costatum* (in % compared to the control). Compounds were tested at 200 µM (Mean ± SD, *n* = 3).

Compound	2 µM	20 µM	200 µM
CuCl_2_	100.7 ± 3.0	101.0 ± 3.9	0 ± 0
ZnCl_2_	101.7 ± 1.9	100.6 ± 0.7	94.3 ± 2.1
Cyclam(C_14_)_2_	105.23 ± 0.6	98.9 ± 0.8	0 ± 0
Cyclam(Bn)_2_	99.0 ± 0.1	96 ± 1.0	0 ± 0
[Zn-cyclen)]Cl_2_	105.3 ± 1.2	95.5 ± 2.1	91.4 ± 5.2
[Zn-cyclam(C_14_)_2_]Cl_2_	104.7 ± 1.5	101.9 ± 0.3	0 ± 0
[Zn-cyclam(Bn)_2_]Cl_2_	99.8 ± 3.1	94.9 ± 3.8	0 ± 0
[Zn-cyclen(C_14_)_2_]Cl_2_	100.7 ± 2.7	93.8 ± 1.6	0 ± 0

## Data Availability

Data are contained within the article. Further inquiries can be directed to the corresponding author.

## Supplementary Materials

The following supporting information can be downloaded at: https://www.mdpi.com/article/10.3390/molecules30112368/s1, Figure S1: Growth kinetics of *V. natriegens* and *V. aestuarianus* at different concentrations of CuCl_2_ and ZnCl_2_ (29 ± 0.1°C).
