# Supplementary material for: Antifouling Properties of N,N′-Dialkylated Tetraazamacrocyclic Polyamines and Their Metal Complexes
Source: Molecules. 2025 May 29;30(11):2368. doi: 10.3390/molecules30112368 (PMC12155641; doi:10.3390/molecules30112368)
Supplement: Supplementary file 1 [file molecules-30-02368-s001.zip › molecules-3650731-supplementary.pdf]

Supplementary Materials

Antifouling Properties of *N,N′*-Dialkylated Tetraazamacrocyclic Polyamines and Their Metal Complexes

Mathieu Berchel [1,*], Dorsaf Malouch [1], Maryline Beyler [1], Maryline Fauchon [2], Yannick Toueix [2], Claire Hellio [2] and Paul-Alain Jaffrès [1]

[1] Laboratoire Chimie Electrochimie Moléculaire Chimie Analytique (CEMCA), UMR 6521, Centre National de la Recherche Scientifique (CNRS), Faculte des Sciences et Techniques, Univ Brest, 6 Avenue Victor Le Gorgeu, 29238 Brest, France
[2] Institut de Recherche pour le Développement (IRD), Ifremer, Laboratoire des Sciences de l'Environnement MARin (LEMAR), Institut Universitaire Européen de la Mer (IUEM), Centre National de la Recherche Scientifique (CNRS), Univ Brest, 29328 Brest, France
* Correspondence: mathieu.berchel@univ-brest.fr

Kinetics of bacterial growth at different concentrations of $ZnCl_2$ and $CuCl_2$

Figure S1. Growth kinetics of V. natriegens and V. aestuarianus at different concentrations of $CuCl_2$ and $ZnCl_2$ (29 ± 0.1°C)

V. natriegens

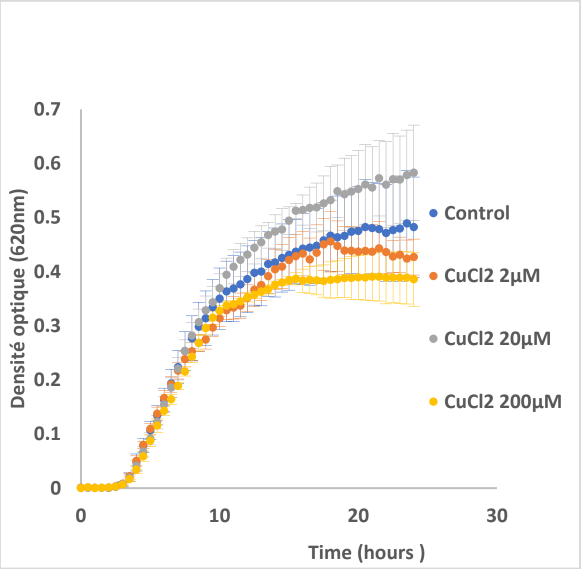

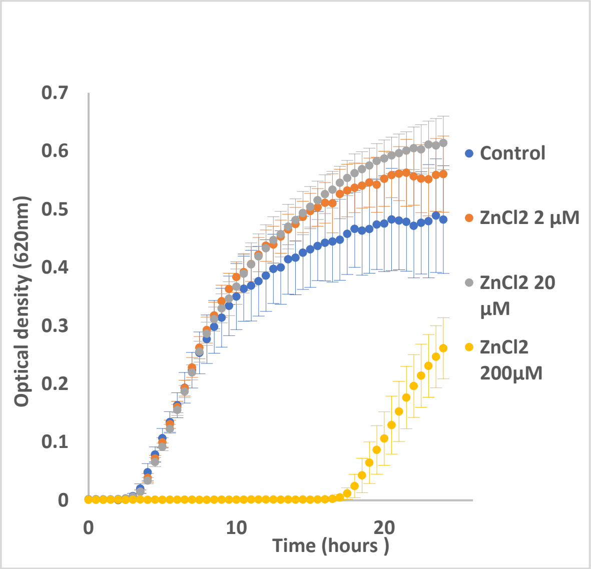

V. aestuarianus

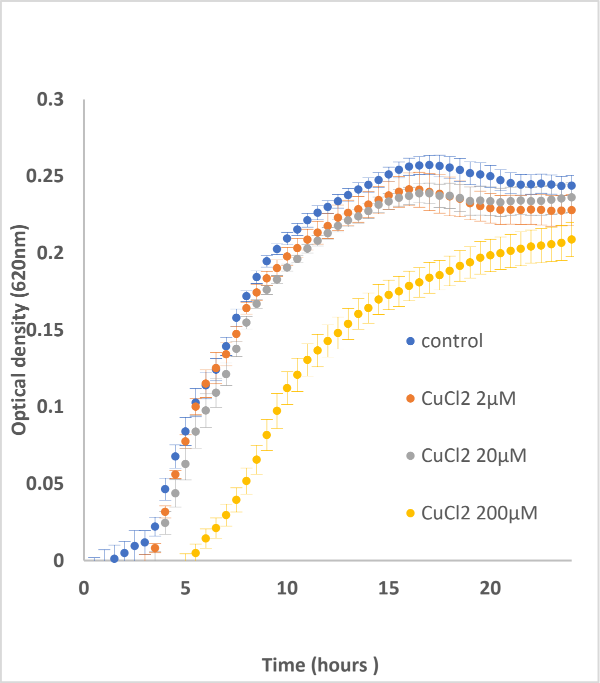
Optical density (620nm)
0.3
0.25
0.2
0.15
0.1
0.05
0
0
5
10
15
20
Time (hours)
control
CuCl2 2µM
CuCl2 20µM
CuCl2 200µM

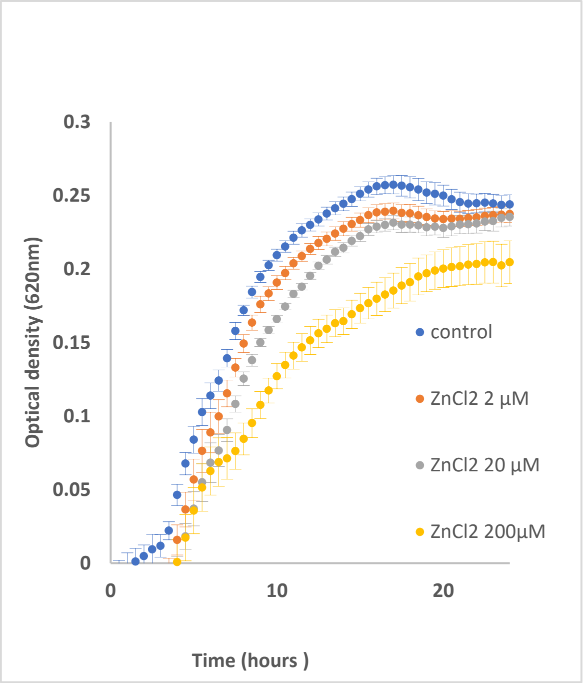
Optical density (620nm)
0.3
0.25
0.2
0.15
0.1
0.05
0
0
10
20
Time (hours)
control
ZnCl2 2 µM
ZnCl2 20 µM
ZnCl2 200µM
